# Where There Is No Paediatric Surgeon!

**Published:** 2011-03-10

**Authors:** Mumtaz Qureshi, Gulfam, Naima Zamir, Jamshed Akhtar

**Affiliations:** Department of Paediatric Surgery, National Institute of Child Health Karachi, Pakistan

**Dear Sir**

Two cases are presented as a preamble to address the issue of management of emergent / urgent paediatric surgical conditions in a setting without paediatric surgical facilities.

**Case 1:**

 A female baby weighing 3 kg referred from a small town near Afghanistan border, after providing initial surgical care for gastroschisis. In privately owned vehicle it takes more than 36 hours to reach our facility from the hospital, where patient was managed initially. The baby was delivered with no antenatal workup. Following birth the patient was immediately transferred to the military hospital in the town where a general surgeon recognized the condition correctly. The referral notes provided details of the condition with clarity. According to the notes impending gangrene of gut protruding out of defect was managed by increasing the defect both in midline and laterally (though incision was too large) with manual stretching of abdominal cavity. The surgeon then applied latex surgical glove after trimming it according to the size of the defect. Neonatal care was provided and the family advised to seek paediatric surgical opinion. On arrival at our hospital the baby was in poor general condition with signs of sepsis. Following resuscitation the baby was re-explored. On removal of silo the color of almost whole of the small bowel was found dusky with doubtful viability patches over the oedematous wall. It was non rotated with jejunal atresia. A large necrotic patch was present near the atretic end (Fig. 1). The terminal part of the atretic gut was resected including necrotic area and end stoma in the left flank made. A silo bag was applied to main defect. The general condition of the baby did not improve and she died on day 5 of admission. 

**Figure:**
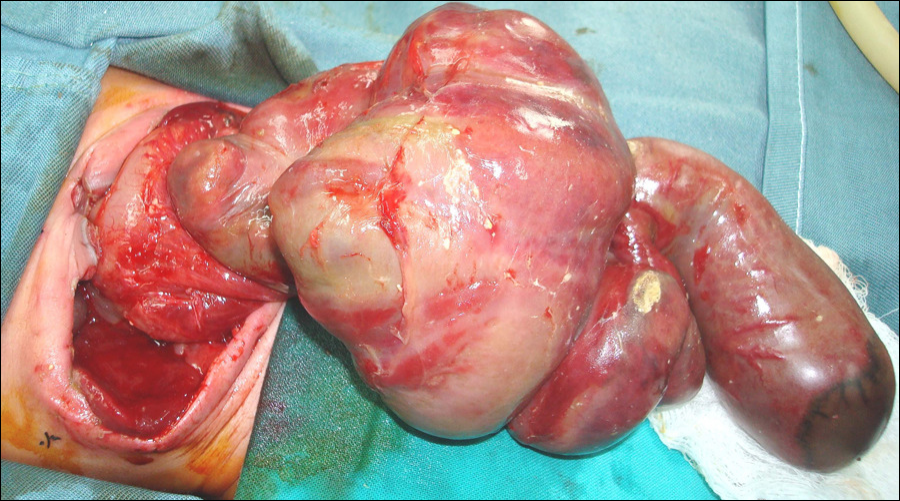
Figure 1: Patient with gastroschisis: Note non rotated atretic small bowel with gangrene and non viable patches over oedematous bowel wall

**Case 2:**

A male newborn following delivery in a small town in rural set up at a government facility, discharged home when after 24 hours parents noted absent anal opening with abdominal distension. The baby was taken to another hospital in the same town where a general surgeon performed surgical procedure for imperforate anus through a large perineal incision under local anaesthesia though an x-ray of baby was also performed. Following surgery, an attempted anoplasty, the baby passed meconium but also started urinating through newly created anal opening; though at birth he passed urine normally per urethra (Fig. 2). The family remained apprehensive and finally brought the patient to our facility. A diverting colostomy was performed and following recovery baby was sent home to be investigated and treated later. At two months of age after investigations, patient was operated through posterior sagittal approach. It was found that the surgeon pulled rectum into perineum while fistulous communication with urinary tract remained intact. In doing so he also transected urethra completely with tissue loss. The fistula was divided and rectum separated from urethra. Both ends of urethra, following identification, excision of scarred tissue, and mobilization of distal end, anastomosed over a silicone catheter. Anoplasty was also fashioned.

**Figure:**
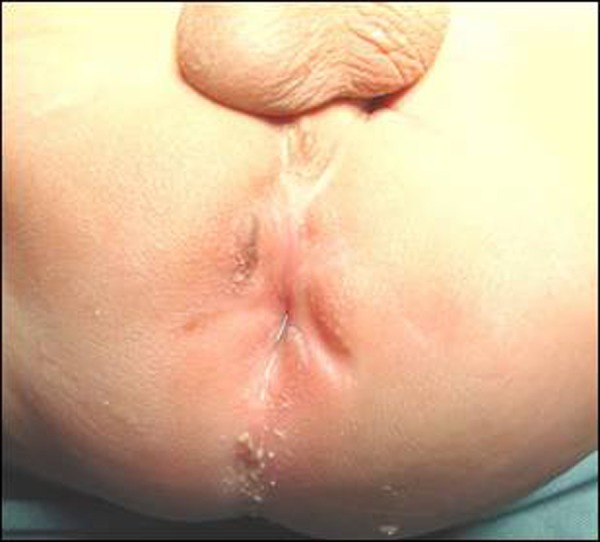
Figure 2: A large irregular scar in the perineum with urine trickling from neo anus

The two cases described above highlight the importance of imparting training to general surgeons in providing safe management to paediatric patients with surgical ailments. Paediatric surgeons are mainly posted / practice in big cities and towns. Thus most of the population can not avail this facility. General surgical facilities on the other hand, are available in most part of the country. Therefore training of general surgeons in order to gain core knowledge related to paediatric surgical conditions, can be a way out of this limitation. As a part of training for fellowship requirement, College of Physicians and Surgeons Pakistan (CPSP) has incorporated rotation of general surgical residents through paediatric surgery but it is not mandatory. The reason being, the non availability of recognized paediatric surgical training institutes in the country. There are limitations to such rotation programs as reported in literature, from countries where it is implemented. The issues are many but the most important being the curriculum and what constitutes adequate exposure and hands on skills attainment. There are also reports on usefulness of such rotation as it does sensitize trainees to the unique needs of paediatric surgical patients [1, 2]. 

Debating further on the issue, the present health care set up in Pakistan is in chaos. Community needs are neither sought nor any plans made to address the deficiency of medical, nursing and paramedical staff in various regions of the country, based upon population statistics and disease pattern. In one of the studies from New Zealand, a detailed assessment was made as to what constitutes paediatric surgical load and what expertise and facilities are offered [3]. In absence of any comprehensive data at government level it thus becomes responsibility of paediatric surgeons themselves to take initiative. A plan must be made to facilitate already qualified general surgeons, for the management of paediatric surgical conditions by holding seminars and workshops at various district levels. These must address what constitutes safe handling of paediatric surgical conditions, what they can offer and how transfer of such a patient to a center with paediatric surgical services be made / facilitated. Only then one can expect reasonable outcome in terms of survival and quality of life of paediatric surgical patients.

## Footnotes

**Source of Support:** Nil

**Conflict of Interest:** None declared

## References

[1]  Skarsgard  ED ( 2009). Does general surgery residency training provide competence in community-based pediatric surgery?. Can J Surg.

[2] Khan  MA,  Hussain  S,  Siddiqui  F (2007). Training general surgery residents in paediatric surgery. J Pak Med Assoc.

[3] Peng  S,  Fancourt  M,  Gilkison  W,  Kyle  S,  Mosquera  D ( 2008). Paediatric surgery carried out by general surgeons: a rural New Zealand experience. ANZ J Surg.

